# Consumer Satisfaction with Telerehabilitation Service Provision of Alternative Computer Access and Augmentative and Alternative Communication

**DOI:** 10.5195/ijt.2015.6180

**Published:** 2015-11-20

**Authors:** EDMUND F. LOPRESTI, ANDREW JINKS, RICHARD C. SIMPSON

**Affiliations:** 1 AT SCIENCES, LLC, PITTSBURGH PA, USA; 2 CENTER FOR ASSISTIVE TECHNOLOGY, UNIVERSITY OF PITTSBURGH MEDICAL CENTER, PITTSBURGH PA, USA; 3 ENGINEERING AND COMPUTING SCIENCES, NEW YORK INSTITUTE OF TECHNOLOGY, OLD WESTBURY NY, USA

**Keywords:** Alternative computer access, augmentative and alternative communication, consumer satisfaction, telepractice

## Abstract

Telerehabilitation (TR) services for assistive technology evaluation and training have the potential to reduce travel demands for consumers and assistive technology professionals while allowing evaluation in more familiar, salient environments for the consumer. Sixty-five consumers received TR services for augmentative and alternative communication or alternative computer access, and consumer satisfaction was compared with twenty-eight consumers who received exclusively in-person services. TR recipients rated their TR services at a median of 6 on a 6-point Likert scale TR satisfaction questionnaire, although individual responses did indicate room for improvement in the technology. Overall satisfaction with AT services was rated highly by both in-person (100% satisfaction) and TR (99% satisfaction) service recipients.

Provision of assistive technology services is often complicated by limited resources. A consumer may not have easy geographical access to rehabilitation professionals with assistive technology expertise (Anderson, Balandin, & Stancliffe, 2014; Theodoros, 2011; Scherer, 2007), particularly if consumers live in more rural areas, have difficulties leaving their homes, or relocate (e.g., for college). Travel to receive assistive technology services can also be complicated by restrictive work or school schedules (Theodoros, 2011). A need to travel to a distant clinic also limits the ability of additional stakeholders (e.g., family members) to participate in assistive technology evaluations. Just as it may be difficult for consumers to travel to a remote clinic, clinical specialists may find it impractical to leave the clinic to travel to distant and rural locations. In some geographical areas there may be a shortage of rehabilitation professionals (ASHA, 2006; Mashima, 2008). Consumers facing external deadlines (e.g., the start of a school semester or training program) may have limited time for equipment trial periods. Consumers with progressive conditions may have rapidly changing motor skills requiring frequent device changes. There is often limited support to update or reconfigure equipment as abilities change (e.g., due to a progressive condition) or as needs change (e.g., due to starting college or a new job).

By all reported measures, the process by which people with disabilities are matched with appropriate computer access technology is not working well enough. Fewer people with disabilities use computers (Dobransky, 2006; Stevenson & McQuivey, 2015). Many people with disabilities who do own computers do not take advantage of computer access technology (Stevenson & McQuivey, 2015). Worst of all, up to a third of computer users who do receive computer access technology abandon it (Federici, 2014; Johnson, Inglebret, Jones, & Ray, 2006; Riemer-Reiss & Wacker, 2000), a statistic which unfortunately has been stable for 30 years despite advances in available assistive technology (Scherer, 2014).

Telerehabilitation (TR) is a valuable clinical service delivery model that includes assessment, therapy, and follow-up services (Brennan et al., 2012). Citing a study by Kairy and colleagues (Kairy, Lehoux, & Vincent, 2009), the World Health Organization affirmed that “telepractice leads to similar or even better clinical outcomes when compared to conventional interventions” (World Health Organization & World Bank, 2011, p. 119), and is a reasonable accommodation to improve service access (World Health Organization & World Bank, 2011). In addition to improving access, TR allows assistive technology services to be delivered in the consumer’s normal home or work environment, which can be more effective than services delivered in a clinic setting (McCue, Fairman, & Pramuka, 2010).

TR has been reported to improve patient access to services, increase cost-effectiveness and efficiency of service provision, and facilitate access to specialist consultation when required (Mashima, 2008). Research has demonstrated videoconference-based TR to be feasible, effective, and appropriate for delivering SLP services at a distance (Brennan, Georgeadis, Baron, & Barker, 2004; Woolf et al., 2015) as long as the auditory and visual transmission is of sufficient quality to allow adequate patient participation and reliable and valid interpretation of signs and symptoms (Duffy, 1997). Research has also demonstrated the feasibility of providing at-a-distance evaluation and therapy services by telephone or closed-circuit television to adults with acquired neurologic speech and language disorders (Duffy, 1997).

The Shepherd Center (Atlanta, GA) has demonstrated the use of TR technology to provide augmentative and alternative communication (AAC) and alternative computer access services (Burns et al., 1998; Burns, Hauber, & Vesmarovich, 2000). In Europe the Remote Service of Rehabilitation Technology (RESORT) project (Panek et al., 2002; Panek & Zagler, Remote Service of Rehabilitation Technology Final Report, 2001) developed TR technology that allowed a clinician to interact with a consumer, monitor computer use, and adjust software settings. AbilityNet in the UK has developed a remote assessment program based on commercially-available communication technologies, including web-cams, Skype and Citrix GoToAssist. A clinician at AbilityNet can initiate a one-to-one connection with a consumer’s PC to demonstrate software on the consumer’s machine, provide training, or make changes to the computer’s operating system (Banes, 2015). AbilityNet has also developed an online self-assessment tool (Banes, 2015).

Significant interest exists amongst SLPs in using TR to overcome barriers of access to services caused by distance, unavailability of specialists and/or subspecialists, and impaired mobility (Mashima, 2008). TR techniques such as interactive web-based virtual environments can enable speech-language pathologists to create highly personalized and engaging activities, increasing consumer motivation such that engagement continues well beyond the therapy sessions (Towey, 2012). In a recent survey, 11% of SLPs and audiologists reported using TR to deliver services but 43% expressed interest in using it in the future (ASHA, 2002). Barriers to adoption of TR included cost (14%), lack of professional standards (13%), lack of data on efficacy and cost-effectiveness (11%), and reimbursement policies (7%) (ASHA, 2002).

In a study of school children, participants receiving speech therapy through video conferencing made similar progress (based on student progress reports and articulation scores) compared with participants receiving in-person speech therapy (Grogan-Johnson, 2010). Further, satisfaction surveys indicated that the students and parents overwhelmingly supported the telemedicine service delivery model (Grogan-Johnson, 2010).

The Oxford Aiding Communication in Education Center has found high satisfaction with TR services such as low-cost videoconferencing and online software sharing in providing assessment, training and support among consumers at remote sites (Donegan, 2002; Lysley, Colven, & Donegan, 1999), and found that TR interventions were perceived to be of higher quality than more conventional communication methods (e.g., phone, email) among participants in an assistive technology loan program (Hazell & Colven, 2001).

While promising work has been conducted in this area, further research and development are needed to discern stakeholders’ perceptions of TR services (e.g., acceptability and viability), identify the limitations and barriers of TR provision, and develop potential solutions (Anderson et al., 2012).

The objective of this study was to determine whether individuals could obtain appropriate prescriptions for computer-based assistive technology through the use of a TR system. We compared an entirely TR-based service delivery program, an entirely in-person program that mirrors current practice in most clinics, and a service delivery program that combines both in-person and TR-based interaction. This paper focuses on consumer perception of the quality and acceptability of their service provision. Other assistive technology outcomes are discussed elsewhere.

## METHODOLOGY

### EQUIPMENT

Video conferencing during study sessions was conducted using a Versatile and Integrated System for Telerehabilitation (VISYTER), developed at the University of Pittsburgh (Parmanto et al., 2010; Saptono, 2008). VISYTER is a web-based TR portal and integrated videoconferencing system based on cost-effective, open-source software. VISYTER has been successfully used to deliver a variety of services including vocational rehabilitation, speech-language therapy, seating and wheeled mobility, and home modifications in a practical and effective manner (Allegretti, Schein, Schmeler, & Brienza, 2011; Kim, Brienza, Lynch, Cooper, & Boninger, 2008; Parmanto, Saptono, Murthi, Safos, & Lathan, 2008; Schmeler, Schein, McCue, & Betz, 2009; Schutte et al., 2012). Of particular interest for this study, VISYTER allows for one site to send multiple video streams. For example, it is possible to transmit a view of the consumer’s face and a second view of the consumer’s hands on a keyboard, or a view of the consumer’s posture and a second view of an AAC device screen.

In computer access and AAC evaluations and training, it is often important to see what is happening on the consumer’s computer or AAC device screen. Screen sharing software (specifically TeamViewer, http://www.teamviewer.com/) was used to allow the clinician to remotely view and control the consumer’s device. TeamViewer also provides a single video stream of each person (i.e., one camera for the consumer and one camera for the clinician) and voice over IP (VOIP).

For AAC devices, use of TeamViewer required that the device be based on a computer running the Windows or Mac operating system and that the device be “unlocked” – that is, it was possible to access the underlying operating system and not just the AAC software. When use of TeamViewer was not possible, VISYTER was used with one of the cameras directed to the device screen.

Hardware on the clinician side included a computer running VISYTER and TeamViewer and a USB speakerphone (ClearOne Chat 60, http://www.clearone.com/). When using a computer that did not have an integrated webcam, a Logitech HD Pro (www.logitech.com) was mounted on top of the monitor. For AAC assessments, whenever possible the clinician had access to AAC devices with similar hardware and/or software to those which would be used for evaluation or training on the consumer side.

Hardware on the consumer side included a computer running VISYTER and TeamViewer, one or more webcams with mounting methods, and a ClearOne Chat 60 USB speakerphone; as well as any assistive technology hardware required for the assessment itself (e.g., AAC devices, alternative mice and keyboards). Webcams available for use during the assessment included two Logitech HD Pro webcams, a Logitech Orbit webcam, and an U-corder (http://www.ucorder.com/). Typically, when using a computer that did not have an integrated webcam, a Logitech HD Pro was mounted on top of the monitor. To view the user’s hands or posture, either (1) a Logitech HD Pro would be mounted to a tripod, (2) a Logitech HD Pro would be mounted to a desk-mounted adjustable arm, or (3) a Logitech Orbit would be located on the desk. To view AAC device screens, a Logitech HD Pro mounted on a tripod would be set to show the view from over the consumer’s shoulder. In place of the tripod-mounted camera, some participants wore an U-corder, a small (13.2 cm × 5.1 cm × 21.8 cm) video camera which can be worn with a lanyard. When the U-corder was worn on the participant’s chest, the video showed a view of the AAC device similar to the participant’s own view. In addition, a mobile wireless hotspot was used to provide Internet access. On occasions when the mobile wireless hotspot did not have a strong signal, the consumer location had Internet access, and the consumer gave permission, the consumer’s Internet access was used. Hardware is shown in **Error! Reference source not found.**.

### RECRUITMENT

Participants were recruited from individuals referred to the University of Pittsburgh Medical Center (UPMC) Center for Assistive Technology (CAT) in Pittsburgh, PA or the Center for Assistive and Rehabilitative Technology (CART) at the Pennsylvania Office of Vocational Rehabilitation’s Hiram G. Andrews Center in Johnstown, PA. Participants were recruited from among consumers referred for AAC or alternative computer access assessments. Involvement of human subjects in this study was overseen by the University of Pittsburgh Institutional Review Board.

Participants were assigned to one of three groups:

Members of the control group received all services with an assistive technology specialist physically present.
Members of the mixed group received an initial assessment with the assistive technology specialist physically present and all remaining services remotely, as described below.
Members of the TR group received all services remotely, as described below.

Sixty-six AAC consumers and 38 computer access consumers were enrolled in the study. Table 1 shows participant completion of the survey instruments described below. The consumer satisfaction survey was introduced as a modification partway through the study, so that many participants completed the study before that survey was introduced. Several participants assigned to the mixed group completed the initial in-person session but discontinued clinical services without any follow-up remote sessions, so that no Telerehabilitation Questionnaires (TRQs) were completed. This was particularly true for computer access clients, who more often discontinued or no longer needed services after the initial evaluation. Participants in the control group by design did not participate in any remote sessions, and so did not complete any TRQs. Participants completed additional outcome measures following their clinical services; these are outside the scope of this paper.

### PROCEDURES

All participants were asked to complete an enrollment form which asked for their name, address, and other contact information. Participants receiving AAC services who had some functional speech and reading ability were assessed on an abridged version of the Assessment of Intelligibility of Dysarthric Speech (Yorkston, Beukelman, & Traynor, 1984). In this assessment, participants first read a word of their selection aloud from a list of 12 words; this was repeated for 10 word lists. Participants then read two sentences of five words in length and two sentences of six words in length. Percentage scores of word and sentence intelligibility were recorded.

Assistive technology assessments followed standard clinical procedures in the CAT and CART. All participants received an initial assessment. Depending on individual needs, this initial assessment was followed by one or more sessions potentially including: one or more training sessions on evaluation equipment in the clinic with follow-up after training; one or more iterations of set-up of loaner equipment and follow-up after a loan period; final evaluation of equipment for the participant; set-up of the participant’s own assistive technology equipment; and/or one or more training sessions on the participant’s own assistive technology equipment. During all sessions, the participant received services from an assistive technology specialist (a speech-language pathologist with AAC expertise or a rehabilitation engineer with alternative computer access expertise).

During remote sessions for the Mixed and TR groups, the assistive technology specialist guided the assessment process remotely by video conference. Another investigator was physically present with the participant to set up the hardware and computer link (VISYTR or TeamViewer) and assist with physically manipulating equipment (e.g., moving cameras, positioning assistive devices, adjusting AAC device settings).

Following each remote TR session, members of the TR and Mixed groups completed a Telerehabilitation Questionnaire (TRQ). The TRQ consisted of seven Likert-style (6-point) questions related to whether the participant was satisfied with the remote assessment process.

Participants in all groups (Control, Mixed, TR) were asked to complete a Customer Satisfaction Survey following each session. This survey include seven yes/no questions related to the participant’s satisfaction with the assistive technology services received, in person or remotely, during that session:

■ The staff was courteous and respectful to you.
■ Services were provided in a prompt and timely manner.
■ You were included in the evaluation or follow-up process and participated in making any decisions regarding your treatment and/or services.
■ The assistive technology devices made available during the course of the evaluation or follow-up were sufficient to assess your needs.
■ The staff answered your questions appropriately.
■ You would recommend the CAT for other individuals with assistive technology needs.
■ Your expectations regarding the evaluation or follow-up were met.

## RESULTS

Telerehabilitation Questionnaires (TRQ) were completed by 38 AAC consumers in the mixed and remote groups. Those participants who did complete the TRQ frequently did so on multiple remote sessions, so that 82 surveys were collected altogether. The TRQ is a Likert-style survey on a scale of 1 to 6, where 1 is labeled “Strongly Disagree” and 6 is labeled “Strongly Agree”. TRQ items are shown in column 1 of Table 2. To evaluate participants’ responses to these questions, the TRQ for each participant’s final session was considered. For these surveys, 95% confidence intervals were calculated across participants; these results are shown in the second column of Table 2.

To evaluate how TRQ responses changed over time, further analyses were performed only for data from the 22 participants who completed more than one TRQ. For these 22 participants, 95% confidence intervals were calculated across the first instance of the TRQ and the last instance of the TRQ. Results are shown in Table 3.

The TRQ was completed by 20 computer access consumers in the mixed and remote groups. Participants frequently completed the TRQ on multiple remote sessions, so that 73 surveys were collected altogether. Similarly to AAC, 95% confidence intervals were calculated across the final surveys for each participant (Table 4).

To evaluate how TRQ responses changed over time, further analyses were performed only for data from the 15 participants who completed more than one TRQ. For these 15 participants, 95% confidence intervals were calculated across the first instance of the TRQ and the last instance of the TRQ. Results are shown in Table 5.

Across all 58 AAC and computer access participants who completed TRQ’s, the median response for each of the seven items was 6.

To further consider changes from the first TRQ to the final TRQ, an ANOVA was performed with factors of time (initial vs. final), subject, and question. Question 3 was not included because multiple participants left this item blank for either their initial or final TRQ. Of the 37 participants who completed at least two TRQ’s (per Table 3 and Table 5), four participants were removed who left multiple questions blank on either their initial or final TRQ. For the remaining 33 participants, the ANOVA results indicated no significant difference between initial and final surveys at the p<0.05 level (p=0.08), a significant effect of which question is being asked (p<0.001), and no significant interaction between question and time. Post-hoc analysis with Tukey’s method indicated that the significant differences were between questions 2 & 6, 2 & 7, 4 & 6, 4 & 7, 5 & 6, and 5 & 7.

Twenty AAC participants completed customer satisfaction surveys. Seven control group participants reported 100% satisfaction; 10 mixed group participants reported an average of 99% satisfaction; and three remote participants reported 100% satisfaction.

Three Computer Access participants completed customer satisfaction surveys, all in the remote group. All three participants reported 100% satisfaction.

## DISCUSSION

Our hypothesis was that the telerehabilitation intervention would be acceptable to participants, demonstrated by TRQ scores significantly at or above 5.0 across items. For the final TRQ for AAC participants (Table 2), this criterion was met for five of seven items. For computer access participants (Table 4), the criterion was only met for items “I was comfortable being evaluated through this means,” “Consulting… through tele-video conferencing saved me monetary expenses….,” and “I would be willing to use this tele-video evaluation process again.”

TRQ responses consistently met our benchmarks for consumer satisfaction for items “I was comfortable being evaluated through this means,” “Consulting with an expert clinician through tele-video conferencing saved me monetary expenses (i.e., travel time, gas, taking off of work, family, etc.),” and “I would be willing to use this tele-video evaluation process again.” Satisfaction scores tended to be lowest for the items “The results of the evaluation through the tele-video conference would be as accurate as an evaluation being completed in-person by a certified practitioner” and “The technology did not interfere with the assessment.” Indeed, the Tukey’s post-hoc test for the pooled AAC and computer access results indicate that participant responses regarding potential savings related to travel and scheduling, and willingness to use the tele-video process, were significantly more positive than their responses regarding the tele-video process being as accurate as in-person services, lack of interference from the technology, and adequate audio and video quality.

These results indicate that participants would find an in-person evaluation to be more reliable, all things considered. Despite technical difficulties they observed advantages to a remote assessment in not having to travel, did not feel that the remote assessment prevented the clinician from considering all areas of their lives, and participants were willing to continue receiving remote services.

Despite these differences, mean responses were above 5 out of 6 for almost all questions for both AAC and computer access, and median responses were 6 out of 6 for all questions for both AAC and computer access.

For AAC participants, two items which did not meet the benchmark for satisfaction after the initial session did meet the benchmark after the final session. For computer access participants, mean responses for all items increased from the initial session to the final session, and two items that did not meet the benchmark after the first session did meet the benchmark after the second session. However, there was no significant difference between the initial and final session.

Overall consumer satisfaction was highly rated by both in-person and TR service recipients on the consumer satisfaction survey. However, the small number of participants who completed this survey, combined with the use of yes/no questions rather than a survey which allowed for more graded responses, reduce the ability to draw conclusions from this survey data. Further work is needed to compare consumer satisfaction with AT across service provision methods.

TR benefits include elimination of consumers’ travel time to clinic, reduced stress of travel for patients with severe disabilities, and increased staff efficiency if staff do not need to spend time traveling and incurring travel costs. Removing the need for travel also improves consumer compliance with appointments, as travel presents a variety of barriers for consumers, increased potential for more follow-up and training, and provided access to caregivers, family and friends who would not typically attend clinic-based sessions. The opportunity to observe someone in their typical physical and social context can profitably inform the evaluation, compared to observing them in a clinical setting. As an additional benefit, the VISYTR software used here allowed the clinician to view a recorded session, which often provided valuable information when the sessions were replayed.

Some difficulties with TR sessions were due to technical limitations. Inconsistent Internet access or low bandwidth could cause delays and interruptions or reduce audio and video quality. Even with a perfect connection, videoconferencing can make it difficult to perceive a social connection with another person, compared to being physically present in the same room. This level of comfort and satisfaction with a video connection was informally observed to vary across individuals for both clinicians and consumers. At times, it is less efficient for a clinician to remotely describe adjustments that need to be made (e.g., to an AAC device or an alternative keyboard set-up), rather than being able to perform adjustments in person. With only a visual contact (and not a physical presence), the expert clinician felt a need to be more engaged visually than he would in an in-person clinic session. Tasks such as adjusting window size to enlarge or diminish camera views during the session, or providing instructions to the consumer and to the assistant while concurrently operating a similar model of the user’s device, added an additional element of multi-tasking for the expert clinician.

The ability of the clinician to view the consumer’s screen can be limited for devices which do not have their own Internet connection. Computer access interventions taking place on a desktop or laptop computer provided consistent access to screen sharing allowing the computer access clinician to see exactly what the consumer was seeing and, with remote control, directly and actively assist with configuration, demonstration, and error correction activities. AAC devices often did not support screen sharing and therefore required attempts to capture the screen on camera and attempts by the expert clinician to mimic the consumer’s activities on a local device of the same model and configuration. This could also be an issue for devices such as smart phones, tablets, and electronic assistants such as digital recorders or alarm wristwatches. These devices did not support screen sharing (although newer versions of some screen sharing software do support some smart phones and tablets).

TR is particularly difficult during initial evaluation sessions, when it would be desirable for the consumer to trial a wide variety of devices which generally will not be available at a remote location. The initial evaluation also typically involved the most intensive assessments of the consumer and the most paperwork (e.g., clinical forms, equipment loan requests). Later training sessions with a loaner device or therapy sessions with a personal device tended to be more efficient. As the patient had already been evaluated in-person, issues of assessing communicative interaction, physical evaluation, and equipment availability had already been addressed. When the patient was seen for the TR session, the expert clinician was able to more effectively interact and interpret patient’s behaviors and communicative style.

In spite of the challenges, and the fact that it does require many sessions using an AAC telerehabilitation approach to achieve a level of comfort and skill in utilizing multiple levels of technology, the opportunity to provide continuing therapy and assistive technology training through TR was viewed by participating clinicians as outweighing the limitations.

In this study, a member of the research team was always present at the consumer site to serve as a remote assistant. During some sessions, this remote assistant had very little role beyond initially establishing the TR connection. However, the remote assistant was frequently active planning the initial placement of equipment, manipulating evaluation equipment, troubleshooting difficulties with the TR connection or camera placement, confirming what the consumer was seeing on the device screen, and assisting with paperwork (clinical forms as well as paperwork specific to the research study). Such assistance tended to be more important during the initial evaluation session, compared with later training sessions on established equipment. Outside the context of a research study, such support might be provided by a local rehabilitation professional or a family caregiver who is comfortable with technology. In a few research sessions, AAC manufacturer representatives were present in the consumer’s home in addition to the remote assistant. Feedback was consistently positive from these vendors regarding the opportunity to have input from an expert clinician and to facilitate the vendor’s ability to serve the consumer. In this study, the remote assistant was frequently a student investigator; and service as a TR remote assistant may be a valuable training experience for students in the rehabilitation professions in a purely clinical application.

## CONCLUSION

Consumers reported high satisfaction with TR services, and similar satisfaction with their overall AT service experience compared with consumers who received exclusively in-person services. These results are promising, given the potential for TR services to reduce travel demands for both consumers and assistive technology professionals and, therefore, potentially expand availability of AT services and especially follow-up services. However, the experience of conducting AAC and alternative computer access TR services in real-world environments revealed limitations in terms of availability of consistent high-bandwidth Internet connections, need for equipment and staff resources at the client location (especially during an initial evaluation), and TR visual and multi-tasking demands on the clinician. TR might be most practical for follow-up sessions after an initial, in-person evaluation, or in situations when a support person with sufficient expertise can be physically present with the consumer. These observations provide areas for future investigation.

## Figures and Tables

**Figure 1:**
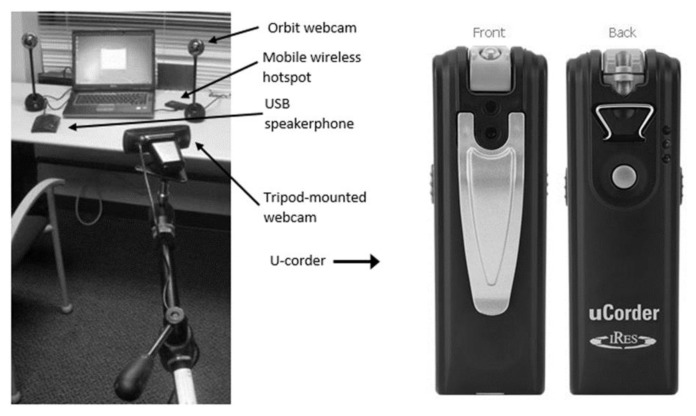
Remote assessment hardware.

**Table 1:** Enrollment Data

		Total Enrolled	Completed TRQ and Consumer Satisfaction Survey	Completed TRQ Only	Completed Consumer Satisfaction Survey Only	No Satisfaction Data Available (see text)
AAC	Control	22	N/A	N/A	7	15
	Mixed	33	8	19	2	4
	Remote	11	3	8	0	0
Computer Access	Control	10	N/A	N/A	0	10
	Mixed	10	0	2	0	8
	Remote	18	3	15	0	0

**Table 2:** TRQ Results for AAC across 38 Participants’ Final Survey Responses. Means and 95% Confidence Intervals

Question	95% Confidence Interval
1. I was comfortable being evaluated through this means.	5.7 (5.2,5.9)
2. The results of the evaluation through the tele-video conference would be as accurate as an evaluation being completed in-person by a certified practitioner.	5.3 (4.9,5.7)
3. All areas of my lifestyle were considered with this process.	5.5 (5.2,5.8)
4. The technology did not interfere with the assessment	5.2 (4.8,5.6)
5. The quality and clarity of the video and audio were acceptable	5.3 (5.0,5.7)
6. Consulting with an expert clinician through tele-video conferencing saved me monetary expenses (i.e. travel time, gas, taking off of work, family, etc.)	5.8 (5.6,6.0)
7. I would be willing to use this tele-video evaluation process again	5.9 (5.8,6.0)

**Table 3:** TRQ Results for AAC Comparing First and Last Instances for 22 Participants Who Completed Multiple TRQs. Means and 95% Confidence Intervals

	First of Multiple Responses	Last of Multiple Responses
1. I was comfortable being evaluated through this means.	5.5 (5.1,6.0)	5.7 (5.4,6.0)
2. The results of the evaluation through the tele-video conference would be as accurate as an evaluation being completed in-person by a certified practitioner.	5.2 (4.7,5.8)	5.4 (5.0,5.8)
3. All areas of my lifestyle were considered with this process.	5.6 (5.2,6.0)	5.4 (5.0,5.9)
4. The technology did not interfere with the assessment	5.5 (4.9,6.1)	5.4 (5.1,5.8)
5. The quality and clarity of the video and audio were acceptable	5.6 (5.2,6.0)	5.4 (5.0,5.8)
6. Consulting with an expert clinician through tele-video conferencing saved me monetary expenses (i.e. travel time, gas, taking off of work, family, etc.)	5.9 (5.7,6.0)	5.9 (5.8,6.0)
7. I would be willing to use this tele-video evaluation process again	5.8 (5.4,6.2)	5.9 (5.6,6.1)

**Table 4:** TRQ Results for Computer Access across 20 Participants’ Final Survey Response

	95% Confidence Interval
1. I was comfortable being evaluated through this means.	5.5 (5.1,5.9)
2. The results of the evaluation through the tele-video conference would be as accurate as an evaluation being completed in-person by a certified practitioner.	5.2 (4.7,5.7)
3. All areas of my lifestyle were considered with this process.	5.3 (4.9,5.8)
4. The technology did not interfere with the assessment	4.8 (4.2,5.5)
5. The quality and clarity of the video and audio were acceptable	5.2 (4.7,5.7)
6. Consulting with an expert clinician through tele-video conferencing saved me monetary expenses (i.e. travel time, gas, taking off of work, family, etc.)	5.5 (5.1,6.0)
7. I would be willing to use this tele-video evaluation process again	5.4 (5.0,5.9)

**Table 5:** TRQ Results for AAC Comparing First and Last Instances for 15 participants Who Completed Multiple TRQs. Means and 95% Confidence Intervals

	First of Multiple Responses	Last of Multiple Responses
1. I was comfortable being evaluated through this means.	5.1 (4.5,5.7)	5.7 (5.4,5.9)
2. The results of the evaluation through the tele-video conference would be as accurate as an evaluation being completed in-person by a certified practitioner.	5.2 (4.6,5.8)	5.4 (4.9,5.8)
3. All areas of my lifestyle were considered with this process.	5.1 (4.4,5.8)	5.3 (4.8,5.9)
4. The technology did not interfere with the assessment	4.9 (4.1,5.6)	5.0 (4.2,5.8)
5. The quality and clarity of the video and audio were acceptable	5.0 (4.4,5.6)	5.2 (4.6,5.8)
6. Consulting with an expert clinician through tele-video conferencing saved me monetary expenses (i.e. travel time, gas, taking off of work, family, etc.)	5.4 (4.8,5.9)	5.7 (5.5,6.0)
7. I would be willing to use this tele-video evaluation process again	5.6 (5.2,6.0)	5.7 (5.3,6.0)
